# Electroacupuncture for insomnia disorder: study protocol for a randomized controlled trial

**DOI:** 10.1186/s13063-017-1922-7

**Published:** 2017-04-13

**Authors:** Sung-Phil Kim, Joo-Hee Kim, Bo-Kyung Kim, Hyeong-Jun Kim, In Chul Jung, Jung Hyo Cho, Jung-Eun Kim, Mi-Kyung Kim, O-Jin Kwon, Ae-Ran Kim, Hyo-Ju Park, Bok-Nam Seo

**Affiliations:** 1 Clinical Research Division, Korea Institute of Oriental Medicine, 1672 Yuseongdae-ro, Yuseong-gu, Daejeon, 305-811 Republic of Korea; 2 Department of Neuropsychiatry, College of Oriental Medicine, Dongeui University, 62 Yangjeong-ro, Pusanjin-gu, Pusan, Republic of Korea; 3 Department of Oriental Gynecology, Jecheon Oriental Hospital of Semyung University, 66 Semyeong-ro, Jecheon, Republic of Korea; 4 Department of Neuropsychiatry, College of Korean Medicine, Daejeon University, 176 Daedukdae-ro, Seo-gu, Daejeon, Republic of Korea; 5 Department of Internal Medicine, College of Korean Medicine, Daejeon University, 176 Daeheung-ro, Jung-gu, Daejeon, Republic of Korea

**Keywords:** Insomnia, Electroacupuncture, Randomised controlled trial, Clinical research protocol

## Abstract

**Background:**

Insomnia is a common sleep disorder that affects many adults either transiently or chronically. The societal cost of insomnia is on the rise, while long-term use of current drug treatments can involve adverse effects. Recently, electroacupuncture (EA) has been used to treat various conditions including insomnia. The objective of this study is to provide scientific evidence for the effect and safety of using EA to treat insomnia.

**Methods/design:**

In this multicentre, assessor-blind, three-arm, parallel-design, randomised controlled trial, 150 participants will be assigned to the EA group, the sham EA (SEA) group, or the usual care group. The EA and SEA groups will receive the specific treatments 2–3 times a week for 4 weeks, for a total of 10 sessions, whereas the usual care group will not receive EA and will continue with usual care during the same time period. The primary outcome measure will be changes in the Insomnia Severity Index 5 weeks after randomisation. The secondary outcomes will include the Pittsburgh Sleep Quality Index, the Hospital Anxiety and Depression Scale, a sleep diary, the EuroQoL-5 dimension questionnaire, the levels of melatonin and cortisol, and the Patient Global Impression of Change. Safety will be assessed at each visit.

**Discussion:**

The results of this multicentre randomised controlled trial will contribute to provide rigorous clinical evidence for the effects and safety of EA for insomnia disorder.

**Trial registration:**

Korean Clinical Trial Registry, CRIS, KCT0001685. Registered on 2 November 2015 (retrospectively registered). Date of enrolment of the first participant to the trial 13 October 2015.

**Electronic supplementary material:**

The online version of this article (doi:10.1186/s13063-017-1922-7) contains supplementary material, which is available to authorized users.

## Background

Insomnia is one of the most common sleep disorders and is defined as a condition in which the patient experiences sleep disturbances accompanied by daytime symptoms or is unable to sleep despite having adequate circumstances and opportunities for sleep [1]. Approximately 40% of adults have experienced some form of insomnia, with chronic insomnia affecting approximately 10–15% of those individuals [2]. According to the Diagnostic and Statistical Manual of Mental Disorders, fifth edition (DSM-5), the diagnostic criteria for insomnia includes difficulty initiating and maintaining sleep, early awakening, and having sleep difficulties at least three times a week and lasting at least 3 months [3]. Furthermore, insomnia refers to a sleep disorder that is not caused by medications, pain, a physical disease, depressive disorder, or other psychiatric illnesses. Insomnia includes not only an inability to fall asleep at night but also an impairment of daytime activities and functioning (i.e. fatigue, nervousness, and a reduction in motivation or energy) with poor concentration, headache, and diminished quality of life [4].

The economic costs associated with insomnia are significant. In the USA, approximately 30–35 billion dollars are spent each year on insomnia, with more than $1253 being spent by the average adult directly and indirectly to manage the condition [5]. Furthermore, given that insomnia is closely linked to depression, anxiety disorders, alcohol or drug abuse/dependence, and suicide, it requires continuous management and treatment beginning in its early stages [6].

Currently, insomnia is most often treated with pharmacological therapy, such as benzodiazepines, and cognitive behavioural therapy. However, while drug treatment can be effective for the short-term management of insomnia, there is little evidence whether efficacy is maintained in the long term, and adverse effects, including feelings of weakness during the daytime, mental slowing, dizziness, sensitivity to light, decreased exercise capacity, and dependency, have been linked to long-term use of these pharmacological agents [7, 8]. Moreover, although randomised controlled trials have reported the efficacy of cognitive behavioural therapy, few therapists are well-trained in this technique for it to be widely used, and there are no clear standardized guidelines about the optimal number and duration of treatments for insomnia [1]. Consequently, treatments for insomnia based on complementary and alternative medicine have received increasing attention.

Acupuncture is now in widespread use, and has been applied for the treatment of various sleep disorders. Recently, several published clinical studies and systematic reviews have shown its use to treat insomnia [9]. According to a systematic review by Yeung et al. [10], 20 randomised clinical trials concluded that acupuncture is more effective than benzodiazepines for the treatment of insomnia; however, these studies have several limitations, including the eligibility criteria used, randomisation, blinding, and small sample sizes. Although several studies have also demonstrated that acupuncture and electroacupuncture (EA) can be effective for treating insomnia [11, 12], poor methodological quality in these studies resulted in a low level of evidence [13]. There have been few multicentre reports on the use of EA for insomnia, and large-scale studies on the efficacy and safety of acupuncture and EA to treat insomnia are necessary. Therefore, the present study aims to provide objective evidence of the effect and safety of EA for insomnia.

## Methods/design

### Study design and setting

**Fig. 1:**
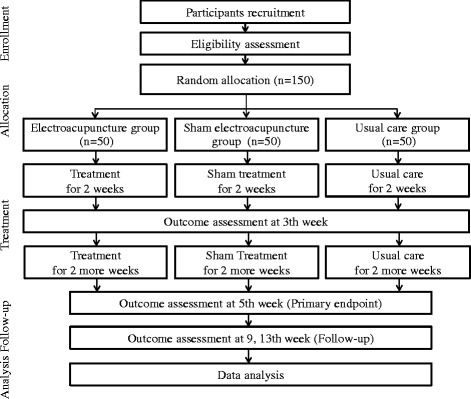
Study flow chart

**Fig. 2:**
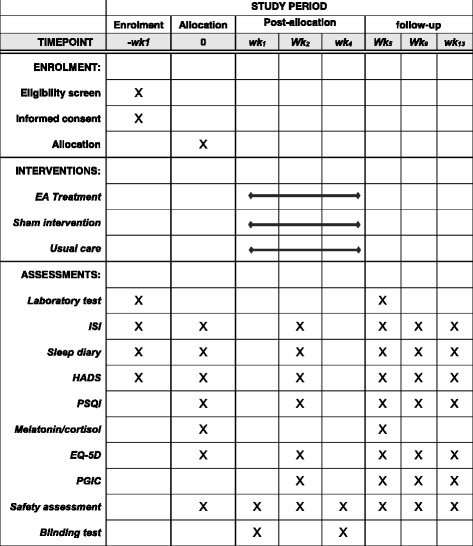
The SPIRIT figure. The schedule of enrolment, interventions, and assessments. *EA* electroacupuncture, *EQ-5D* EuroQoL five dimension questionnaire, *HADS* Hospital Anxiety and Depression Scale, *ISI* Insomnia Severity Index, *PGIC* Patient Global Impression of Change, *PSQI* Pittsburgh Sleep Quality Index

We designed a multicentre, randomised, assessor-blind, controlled, parallel-group study to compare the effect and safety of EA, sham electroacupuncture (SEA), and usual care in subjects with insomnia. The participants will be recruited using regional newspapers, flyers, advertisement boards, etc. Potential candidates for the study will be screened and fully informed about the study. Eligible participants will be randomly assigned to three groups and receive treatment for 4 weeks with 2 months of follow-up. The study flow chart is shown in Fig. 1. The Standard Protocol Items: Recommendations for Interventional Trials (SPIRIT) checklist and figure are given in Additional file 1 and Fig. 2, respectively.

An independent researcher that is blinded to the group allocation will perform the outcome assessment and statistical analysis.

### Study population

#### Inclusion criteria

Candidates who meet the following criteria will be included:

1. age 19–64 years;
2. an Insomnia Severity Index (ISI) score ≥15 points;
3. fulfilment of the DSM-5 diagnostic criteria for insomnia disorder; and
4. willingness to sign an informed consent.

#### Exclusion criteria

Individuals meeting any of the following criteria will be excluded:

1. a diagnosis of major depressive disorder, anxiety disorder, panic disorder, or other psychiatric disorders; caffeine, alcohol, or drug addiction; or a Hospital Anxiety and Depression Scale (HADS) score ≥11 points;
2. working shifts or changes in day/night work schedule that could impact circadian rhythm;
3. suffering from pain severe enough to cause sleep disturbance or presence of any disease that could cause insomnia;
4. having undergone any therapy or taken any medication as required in the previous 2 weeks, or a change in the type or dosage of a regularly taken medication in the previous 4 weeks to alleviate insomnia;
5. taking medication for a cardiovascular or haemostatic disorder;
6. a diagnosed unregulated hormone disorder, serious or systemic disease (e.g. hyperthyroidism, chronic liver disease or chronic kidney disease) that could affect sleep;
7. a previous hypersensitivity reaction to acupuncture or difficulty co-operating with acupuncture therapy;
8. the presence of implants that could interfere with EA or a history of hypersensitivity to electrostimulation;
9. pregnancy, lactation, or planning a pregnancy; and
10. difficulty complying with the treatment, questionnaire, or study protocol

### Randomisation, allocation concealment, and blinding

Subjects who meet the eligibility criteria will be randomly allocated to one of three groups in a 1:1:1 ratio through stratified block randomisation based on whether medications are taken to treat insomnia and the institutions conducting the trial. An independent statistician (OJK) will use SAS® version 9.4 software (SAS Institute Inc., Cary, NC, USA) to generate random assignment tables. The randomisation list will be sealed in sequentially numbered opaque envelopes and delivered to each institution, where they will be stored in a double-locked cabinet. Only the practitioners will be aware of the group allocation of each patient. After random assignment, the envelope will again be stored separately in a double-locked cabinet. Allocation concealment will not be broken until the trial is complete. Since it would be impossible to blind the practitioners, they will not be involved in the assessment. The outcome assessors and data analysts will be blinded to the group allocation.

### Interventions

The EA group will be treated with 0.25 × 40 mm disposable sterilized filiform needles (Dong-bang Acupuncture Inc., Seoul, Korea) applied to the Baihui (GV20), Yintang (EX-HN3), bilateral Shenmen (HT7), Neiguan (PC6), Jinmen (BL63), and Dazhong (KI4) acupoints by inserting and manipulating the needle to achieve *de qi* (2–3 times a week for 4 weeks, for a total of 10 sessions). After *de qi* is achieved, an EA device (ES-160, Ito Co. Ltd., Tokyo, Japan) will be used to apply 4 Hz for 30 min at 80% intensity of the stimulation felt by the participant.

For the SEA group, a Park Sham Device (PSD; Dong-Bang AcuPrime Ltd., Exeter, UK) will be used to needle 10 specific non-acupoints on both the arms and legs during the same period using the same needling schedule as the EA group. The PSD is one of many sham acupuncture methods used in acupuncture studies [14, 15]. It has a blunt end that retracts to shorten the length of the needle and give the appearance that needling will be performed without the needle actually penetrating the skin. Electrostimulation in the SEA group will be performed using the same EA device (ES-160) that is used in the EA group. The de-activated device will be connected to the PSD and applied for 30 min, while making the same beeping sound and light indicators when operated without delivering any electrostimulation.

The EA and SEA groups will be prohibited from undergoing any additional treatments for insomnia during the study period. The planned acupuncture treatment is based on Standards for Reporting Interventions in Clinical Trials of Acupuncture (STRICTA) and Consolidated Standards of Reporting Trials (CONSORT), and detailed information is summarized in Table 1.

**Table 1:** Details of acupuncture treatment based on the revised STRICTA checklist

	Item	Detail
1. Acupuncture Rationale	1a) Style of acupuncture	EA based on traditional medicine theory
	1b) Reasoning for treatment provided, based on historical context, literature sources, and/or consensus methods, with references where appropriate	Textbook on acupuncture and Moxibustion, related literature [10–12, 43], consensus by experts in acupuncture and insomnia
	1c) Extent to which treatment was varied	Standardized treatment
2. Details of acupuncture	2a) Number of needle insertions per subject per session	Fixed 10 acupoints
	2b) Names of points used	EA treatment group: Unilateral GV20, EX-HN3, bilateral HT7, PC6, BL63, KI4
	2c) Depth of insertion	From 5 to 20 mm
	2d) Response sought	*de qi* during needling and perception of stimulus during electrostimulation
	2e) Needle stimulation	After manual stimulation, 4 Hz will be conducted and stimulation will be at 80% intensity of the stimulus the participant perceives
	2f) Needle retention time	30 min
	2 g) Needle type	0.25 × 40 mm sterilized stainless steel needle
3. Treatment regimen	3a) Number of treatment sessions	Total 10 sessions
	3b) Frequency and duration of treatment sessions	2–3 sessions per week for 4 weeks
4. Other components of treatment	4a) Details of other interventions administered to the acupuncture group	All three groups will be educated through sleep hygiene brochure. Usual care group will be permitted to have any type of treatment
	4b) Setting and context of treatment, including instructions to practitioners, and information and explanations to patients	The practitioner will limit unnecessary conversation that does not pertain to the treatment or patient
5. Practitioner background	Description of participating acupuncturists	Korean Medical Doctors who have a license and at least 2 years of clinical experience
6. Control or comparator intervention	6a) Rationale for the control or comparator in the context of the research question, with sources that justify this choice	Park sham placebo device will be used as a sham control [12, 14]
	6b) Precise description of the control or comparator. If sham acupuncture or any other type of acupuncture-like control is used, provide details as for items 1–3 above.	SEA control group: use Park sham placebo device on 10 non-acupoints on both upper and lower limb and connect the electrostimulator in switch-off state for removal after 30 min

*EA* electroacupuncture, *STRICTA* Standards for Reporting Interventions in Clinical Trials of Acupuncture

The usual care group will be allowed to undergo any treatment for insomnia except traditional medicine such as acupuncture, moxibustion, and herbal treatment. The participants will be asked to inform the research investigators of any new treatments received after entry into the trial, and all concomitant treatments will be recorded on the case report form (CRF). All three study groups will be educated using a brochure on sleep hygiene.

### Sample size

The objective of this clinical trial is to conduct a comparative analysis on the effect and safety of EA for treating patients with insomnia. We used data from two previous studies [11, 12] to estimate the difference in the mean ISI values for the EA treatment and SEA control groups as 4.15, and the standard deviation as 6.0. Then the sample size was calculated using a two-sided 2.5% significance level and 80% statistical power. Moreover, the usual care group will require a smaller sample size because differences in the ISI before and after treatment are expected to be larger between the EA and usual care groups than between the EA and SEA groups; therefore, this trial will assign participants to the three groups in a 1:1:1 ratio. Each group will have a minimum of 40 participants for a total of 120 participants, which will allow for a dropout rate of 20%, thus requiring a minimum of 150 participants.

### Outcome measures

#### Primary outcome

The primary outcome measure in this study is ISI, which is a seven-item questionnaire designed to determine the diagnosis and degree of insomnia. The reliability and validity of this questionnaire is well established [16], and the validated Korean version of the ISI will be used in this study [17]. The total score of the ISI ranges from 0 to 28 points, and the participants will be classified as follows: no clinically significant insomnia (0–7 points), subthreshold insomnia (8–14 points), clinical insomnia of moderate severity (15–21 points), and severe clinical insomnia (22–28 points) [17].

#### Secondary outcomes

Sleep quality will be assessed using the Pittsburgh Sleep Quality Index (PSQI) questionnaire. The PSQI is an instrument that assesses sleep quality and disturbance within the past month by measuring seven categories: subjective sleep quality, sleep latency, sleep duration, habitual sleep efficiency, sleep disturbances, use of sleeping medication, and daytime dysfunction. The PSQI has been shown to be highly consistent with sleep diary and polysomnography (PSG) results in patients with primary insomnia, and is recommended as an assessment tool in patients who are suspected of having insomnia [18, 19].

Sleep diaries, which are a daily record on the patient’s sleep, are widely used to collect direct information on sleep and waking patterns in patients with insomnia [20]. In this study, the sleep diary will contain details on when the participant went to bed at night, waking time in the morning, sleep latency, the number of times and duration the participant woke during the night, whether daytime naps were taken, and whether the participant used sleeping aids.

The HADS consists of 14 items with seven odd-numbered anxiety subscales (HADS-A) and seven even-numbered depression subscales (HADS-D). This questionnaire is a validated and reliable psychological instrument that is widely used to assess changes in emotional state, such as anxiety and depression, in patients with chronic diseases. Assessment will be performed using a four-point scale (0–3 points), with higher scores indicating more severe symptoms. Zigmond and Snaith grouped each subscale as follows: non-cases (0–7 points), doubtful cases (8–10 points), and cases (11–21 points) [21]. A previous standardization study reported the sensitivity and specificity of the Korean versions of the HADS-A and HADS-D [22].

To observe physiological changes in the patient, the levels of salivary melatonin and cortisol will be assessed. Melatonin is secreted from the pineal gland and has a direct effect on sleep structure [23]. Cortisol is produced in the adrenal cortex and its secretion is regulated by the hypothalamic-pituitary-adrenal axis. Studies have shown that changes in cortisol concentrations are associated with stress and sleep [24]. Because both melatonin and cortisol concentrations can fluctuate according to a circadian rhythm, the samples will be collected and analysed after scheduling the participant visit and conducting educational sessions on the use of the saliva collection kit in advance [25].

The EuroQoL five dimension questionnaire (EQ-5D) will be used to measure health-related quality of life. This instrument is designed to have the subject check the most appropriate response to five items on health status; each checked item will have a weighted score of 1, 2, or 3, and the combination of the five numbers will represent the health status of the subject [26]. In addition, the EQ-5D and ISI will be used to estimate the parameters needed for economic evaluation. The Patient Global Impression of Change (PGIC) will be used to assess the participant’s subjective perception of overall improvement after treatment.

The time points for each assessment and the procedures are summarized in Table 2.

**Table 2:** Schedule for treatment and outcome assessment

Visit	Screening	1	2–5	6	7–10	11	12	13
Week		1	1–2	3	3–4	5	9	13
Informed consent	●							
Inclusion/exclusion criteria	●							
Treatment expectation questionnaire	●							
Vital signs	●	●	○	●	○	●	●	●
Demographic characteristics	●							
Medical history	●							
Laboratory tests	●					●		
Random allocation		●						
Change of medical history		●	○	●	○	●	●	●
EA treatment		○	○	○	○			
ISI	●	●		●		●	●	●
Melatonin/cortisol study		●				●		
PSQI		●		●		●	●	●
Sleep diary	●	●		●		●	●	●
HADS	●	●		●		●	●	●
EQ-5D		●		●		●	●	●
PGIC				●		●	●	●
Blinding test		○			○			
Safety assessment		●	○	●	○	●	●	●

●, All groups; ○, treatment groups

*EA* electroacupuncture, *EQ-5D* EuroQoL five dimension questionnaire, *HADS* Hospital Anxiety and Depression Scale, *ISI* Insomnia Severity Index, *PGIC* Patient Global Impression of Change, *PSQI* Pittsburgh Sleep Quality Index

### Statistical analysis

The primary outcome measure in this study is the mean change in the ISI from baseline to week 5. A two-sided test with a significance level of 0.025 will be performed using an analysis of covariance with baseline as the covariate and the treatment group as the fixed factors. The intra-group ISI changes from baseline to post-treatment will be analysed using Student’s paired *t* test or Wilcoxon signed-rank test, and reported with a 95% confidence interval. In addition, we will use a repeated-measures analysis of variance to identify any trend changes. Dunnett’s test will be used for inter-group comparisons to compensate for multiple comparisons.

The methods used to analyse the secondary outcome measures, i.e. ISI at weeks 3, 9, and 13, PSQI, HADS, EQ-5D, PGIC, changes in sleep diary entries, and changes in melatonin and cortisol levels, will be the same as those used for the primary assessment analyses. The chi-square test or Fisher’s exact test will be used for categorical data. Analyses will be conducted on an intent-to-treat basis, and multiple imputations will be applied if there are missing values.

### Data handling and safety monitoring

All data will be collected in compliance with the approved protocol and will be recorded on a CRF. All adverse events that are not necessarily related to the treatment will be observed and reported by the participants and researchers at each visit. In the event of serious adverse reactions, detailed reports will be drafted and assessments will be made based on the protocol. Blood tests, including complete blood count, differential count, and renal and liver function tests, will also be performed at the screening visit and after the end of treatment. Data and safety monitoring will be conducted at all sites periodically during the study.

## Discussion

This study is a multicentre randomised controlled clinical trial with an appropriate sample size on the effect and safety of EA for treating patients with insomnia disorder according to the criteria of the DSM-5.

The differentiation of primary and secondary insomnia which existed in the previous sleep disorder diagnosis systems was excluded in the DSM-5 diagnostic system [27]. There is currently no conclusive causal relationship or direction between sleeplessness and other co-existing medical or psychiatric illnesses [27]. Insomnia does not simply mean experiencing difficulty sleeping during the night, and it has a considerable negative impact on various aspects including daytime functioning, emotion, quality of life, and socio-economic burden. Therefore, it is important to make a comprehensive assessment of all these domains in clinical trials that evaluate the therapeutic effect on insomnia [28].

In the present study, ISI and PSQI will be used to assess the insomnia symptom severity, the chief complaints of insomnia patients. The ISI was selected as the primary outcome to assess the effects of EA on insomnia because it can directly assess the severity of insomnia, disruption to quality of life, and interference with daytime activities over the past 2 weeks. A sleep diary will be evaluated as a sleep/wake parameter in addition to the questionnaires. The HADS will be used to assess the psychological symptoms such as anxiety and depression, which are commonly associated with insomnia patients. The PGIC will be used as a global assessment outcome measurement. Quality of life will be evaluated using the EQ-5D. Moreover, there are several reports indicating patients with sleep disorders use more health care resources [29, 30]. As a result, an additional economic evaluation will be conducted to investigate the cost-effectiveness of electroacupuncture treatment for insomnia disorder.

EA has been used in various clinical conditions, including insomnia and psychiatric disorders. However, the mechanisms underlying the effect of EA have not yet been elucidated. A study by Tang et al. reported that low-frequency EA is an effective treatment for post-stroke insomnia and can regulate the level of neurotransmitters, including 5-hydroxytryptamine and norepinephrine in patients with post-stroke insomnia [31]. Additionally, insomnia has been shown to be related to changes in melatonin and cortisol levels, and several previous studies reported the efficacy and safety of using melatonin to treat insomnia [32–34]. Melatonin is a hormone secreted from the pineal gland in the brain and is important for regulating the sleep/wake circadian rhythm. In general, melatonin is secreted at high levels immediately before sleep and during the night, but its serum concentration decreases during the daytime and activity. Nordio et al. assessed the 24-h urinary melatonin metabolite rhythm and suggested that acupressure at the H7 acupoint may involve regulation of melatonin [35]. Spence et al. also reported that acupuncture increased the nocturnal endogenous secretion of melatonin and improved polysomnographic measures [36]. Cortisol is closely associated with the stress response and can cause insomnia when produced in excessive amounts [24]. Previous studies using animal models have reported that GV20, EX-HN3, and HT7 needling or EA stimulation can suppress the secretion of cortisol [37, 38]. In the present study, the levels of melatonin and cortisol will be analysed before and after treatment to explore the physiological mechanism of EA in the treatment of insomnia.

There are limitations to this study. First, we will perform sham electroacupuncture using a sham EA device and PSD as a control intervention in the SEA group. However, the currently developed sham acupuncture interventions are not completely inert physiologically, and previous studies reported that sham acupuncture interventions not only have some physiological activity, but could also be associated with larger effects than pharmacological and other physical placebos [39–41]. Placebo effects are very commonly observed and should be controlled appropriately in sleep disorder clinical trials [42]. Therefore, we will apply the non-penetrating placebo device at non-acupoints to minimize this bias, and we will also include a usual care group as a control. Second, although PSG is generally considered the gold standard in sleep studies, it is not always included in actual clinical trials due to its costs, burden on patients, and research conditions. In the present multicentre trial, PSG is not feasible and various outcome measures including a sleep diary, ISI, PSQI, HADS, melatonin and cortisol will be assessed. We will evaluate objective sleep parameters such as PSG or actigraphy in future studies. In spite of its limitations, the results of this multicentre randomised controlled trial may provide rigorous evidence for a multi-faceted evaluation of the effect and safety of EA for treating insomnia.

### Trial status

This clinical trial received Institutional Review Board approval and is currently recruiting participants.

## Additional file

Additional file 1: SPIRIT checklist. (DOC 121 kb)

## Abbreviations

CONSORT: Consolidated Standards of Reporting Trials
CRF: Case report form
DSM-5: Diagnostic and Statistical Manual of Mental Disorders, fifth edition
EA: Electroacupuncture
EQ-5D: EuroQoL five dimension questionnaire
HADS: Hospital Anxiety and Depression Scale
ISI: Insomnia Severity Index
PGIC: Patient Global Impression of Change
PSG: Polysomnography
PSQI: Pittsburgh Sleep Quality Index
SEA: Sham electroacupuncture
STRICTA: Standards for Reporting Interventions in Clinical Trials of Acupuncture
